# THE MULTI-DIMENSIONAL ROLE OF GRATITUDE IN FAMILY MEMBERS OF PEOPLE WITH EARLY-STAGE ALZHEIMER’S

**DOI:** 10.1093/geroni/igad104.3751

**Published:** 2023-12-21

**Authors:** Jocelyn McGee, Davie Morgan, Rebecca Meraz

**Affiliations:** Baylor University, Waco, Texas, United States; Baylor University, Waco, Texas, United States; Baylor University, Waco, Texas, United States

## Abstract

The interest in and scientific understanding of the multifaceted construct of gratitude has grown in recent years. However, research on the role gratitude plays in the lives of family members of persons with early-stage Alzheimer’s disease is limited. The purpose of this study, therefore, was to uncover the ways gratitude is experienced and expressed in family members of persons with early-stage Alzheimer’s. Interpretative phenomenological analysis, a qualitative methodology, was employed to analyze the narratives of 27 family members. Expressions of gratitude were universal in the study but varied in the dispositional gratitude factors of frequency, intensity, span, and density. Relational conceptions of gratitude were expressed across three subthemes: (1) intrapersonal (e.g., family member grateful for how their own personal strengths and resources had aided them and allowed for growth while caring for their loved one with early-stage Alzheimer’s; family member grateful for the individual strengths their family member with early-stage Alzheimer’s had retained); (2) interpersonal (e.g., family member expressed gratitude for people who had positively influenced them such as prior role models for giving care to a person with Alzheimer’s and people who had shown the family compassion); and 3) transpersonal (e.g., gratitude for God or a divine entity or force that was deemed benevolent and helpful when it came to the experience of living with Alzheimer’s). Findings indicate that professionals should attend to gratitude in family members of persons with Alzheimer’s and leverage this multifaceted resource to improve health and well-being.

